# Embolization for intractable spontaneous hemarthrosis of the knee joint in the elderly patient: Case report

**DOI:** 10.1097/MD.0000000000020475

**Published:** 2020-06-12

**Authors:** Suk Kyoon Song, Seung Bum Chae, Dae Won Kang, Won Kee Choi

**Affiliations:** Department of Orthopaedic Surgery, College of Medicine, Daegu Catholic University, Daegu, Korea.

**Keywords:** Angiogram, elderly, hemarthrosis, spontaneous

## Abstract

**Rationale:**

Several case reports about the diagnostic and therapeutic approaches of hemarthrosis after total knee arthroplasty using angiogram have been reported, owing to the probability of bleeding caused by vascular injuries. However, there were only few cases of spontaneous hemarthrosis of the knee joint in the elderly patient that have not undergone total knee arthroplasty that have been previously reported.

**Patients concerns:**

An 82-year-old male presented to our outpatient department with acute left knee pain. He had no history of trauma. The patient had under gone several times of therapeutic arthrocentesis for treatment of left knee joint effusion at a local clinic.

**Diagnosis:**

Arthroscopic examination was performed at the local clinic and was not able to reveal any focus of intra-articular bleeding. We consulted this case with the department of radiology to angiographically find out abnormalities of the genicular arteries. Angiographs showed hyper vascularity of the superior and inferior lateral genicular artery, and superior medial genicular artery.

**Intervention:**

One-step embolization using micro-catheter and 50 to 150 μm gelfoam particles was conducted. The hypervascular findings shown on angiogram were markedly subsided after embolization.

**Outcomes:**

Until 1 year after embolization, there were no signs of recurrence on outpatient follow-up sessions

**Lessons:**

Degenerative changes of the genicular arteries may be a cause of spontaneous knee joint hemarthrosis in the elderly patients. Angiographic diagnosis and treatment may be effective for such cases.

## 1 Introduction

Spontaneous hemarthrosis of the knee joint is very rare in the elderly population.^[1]^ Causes include subchondral bone bleeding, lateral meniscus injury, genicular artery bleeding, and the use of anticoagulants.^[1–3]^ Various treatment options, from conservative management with bed resting to surgical managements such as arthroscopic procedures, have been reported. Several case reports about the diagnostic and therapeutic approaches of hemarthrosis after total knee arthroplasty using angiogram have been reported, owing to the probability of bleeding after vascular injuries.^[4,5]^ However, cases of spontaneous knee joint hemarthrosis in the elderly patient who have not undergone total knee arthroplasty have been rarely reported. We hereby report a case of spontaneous hemarthrosis of the knee, which was thought to be caused by degenerative changes in the genicular artery, and which was managed by 1-step embolization of the hypervascular lesion found on angiogram.

Informed written consent was obtained from the patient for publication of this case report and the accompanying images.

## 2 Case

**Figure 1:**
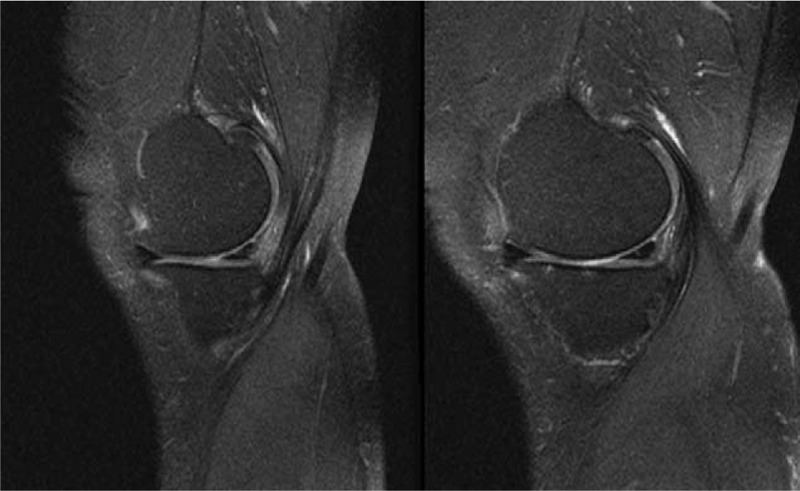
MRI showed degenerative changes in the medial meniscus.

An 82-year-old male presented to our outpatient department with acute left knee pain. He had no history of trauma. Before he visited our hospital, he had undergone several times of therapeutic arthrocentesis for treatment of left knee joint effusion at a local clinic. The patient has recalled that all the aspirates to be bloody. He had been taking antiplatelet agents for 2 years, after percutaneous coronary intervention. On presentation, there were no visible joint effusion in both knees, and he was wearing a knee immobilizer. After consultation with the cardiology department, we recommended the patient to discontinue the antiplatelet agent for a week and to take a rest for a while. A month after his initial presentation, the patient visited our emergency department with the same left knee pain. After experiencing left knee pain the day right after his initial OPD visit, he visited another local clinic and was recommended for MRI testing, followed by arthroscopic examination and synovectomy. There were no definite findings of meniscal tear but only degenerative changes in the medial meniscuson MRI (Fig. 1). Despite arthroscopic procedures, his left knee joint hemarthrosis showed no improvement, and as a result, the patient was transferred to our emergency department from the local clinic.

**Figure 2:**
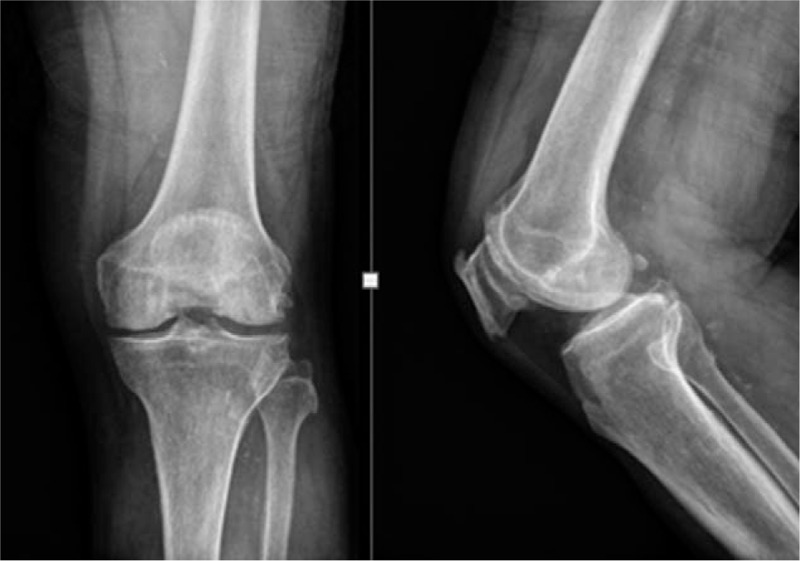
X-ray showed mild arthritic changes and no joint effusion.

**Figure 3:**
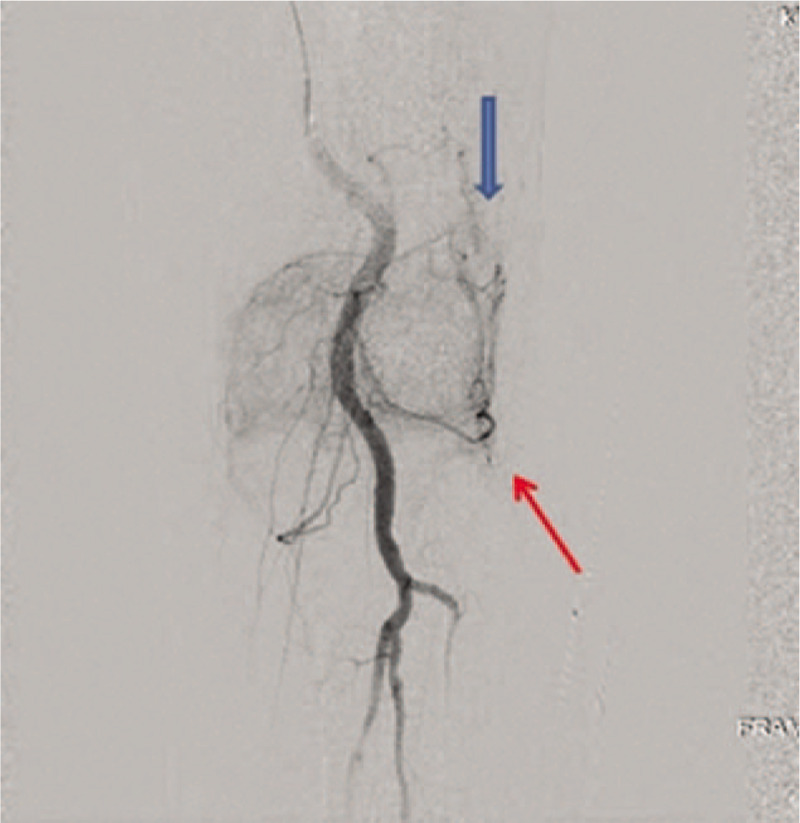
Angiographs revealed hypervascular tissue staining of the superior (blue arrow) and inferior lateral genicular artery (red arrow) and superior medial genicular artery.

**Figure 4:**
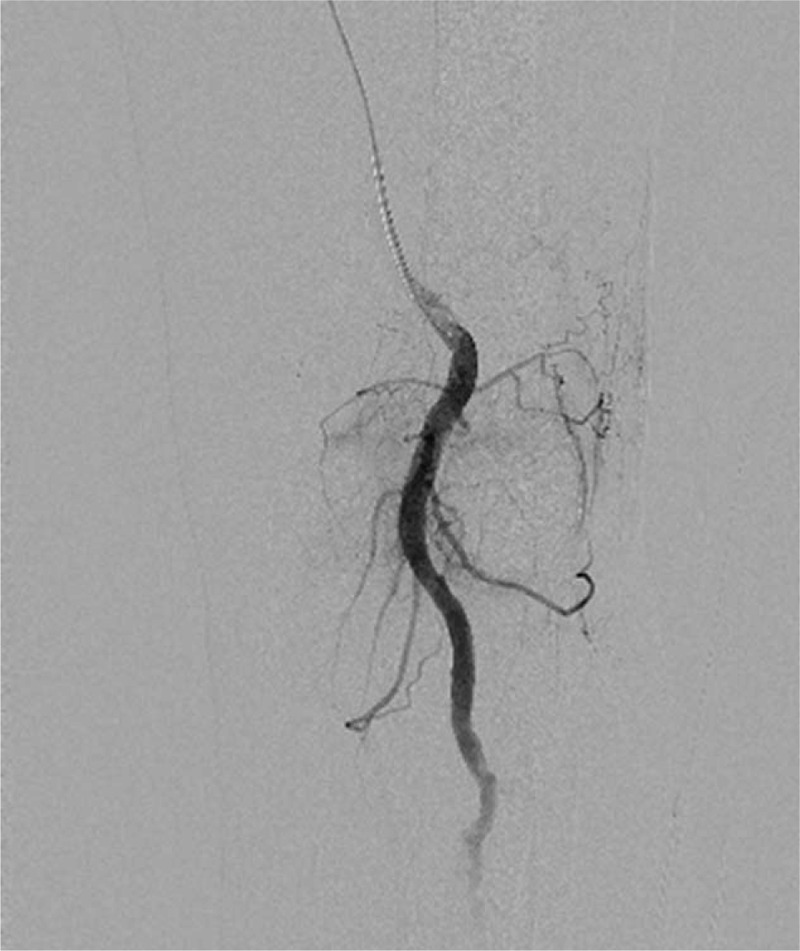
The hypervascular tissue staining was markedly subsided after embolization.

On presentation, the patient showed moderate effusion on his left knee (Fig. 2), with resting pain. After excluding the diagnosis of infection by joint fluid analysis, we consulted with the cardiology department about the matter and decided to hold the antiplatelet agent. Although his degree of knee joint effusion showed improvement after 4 days of bed rest, knee pain on rest continued. No focus of intra-articular bleeding was revealed on arthroscopic examination, which was performed at the local clinic. We consulted this case with the department of radiology to find out any abnormalities of the genicular arteries angiographically. Angiographs revealed hypervascularity of the superior and inferior lateral genicular artery and the superior medial genicular artery (Fig. 3). One-step embolization was conducted using microcatheter and 50 to 150 μm gelfoam particles. The hypervascular lesions markedly subsided after embolization(Fig. 4).

**Figure 5:**
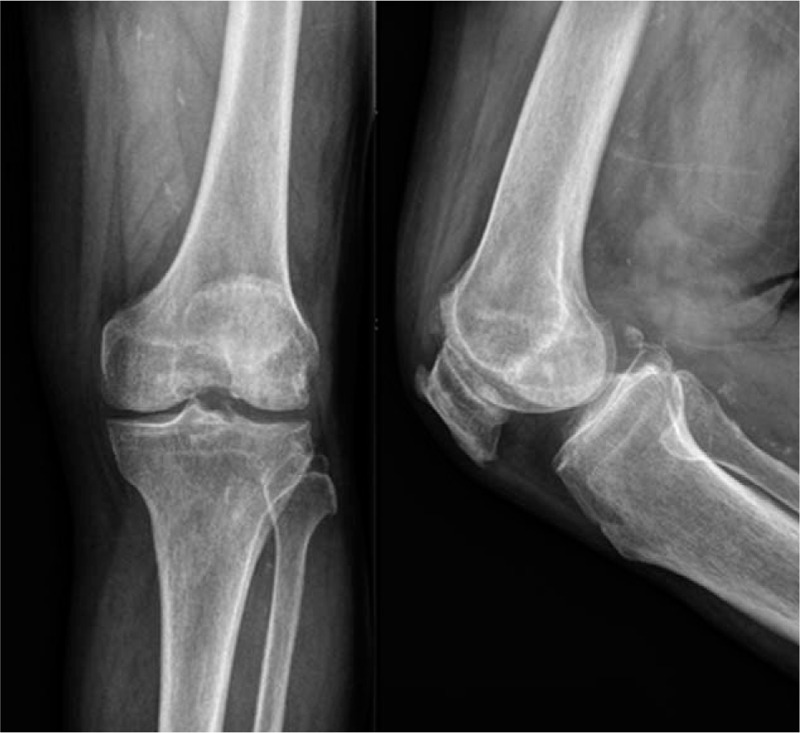
There were no signs of recurrence on outpatient follow-up X-ray until 1 year after embolization.

After embolization and 3 days of bed rest, the patient was permitted for partial weight bearing. We closely monitored for the relapse of symptoms during the 3 days after partial weight bearing. After confirming that no symptoms relapsing for 3 days, we decided to discharge the patient and to resume the antiplatelet agent. There were no signs of recurrence on outpatient follow-up sessions until 1 year after embolization (Fig. 5).

## 3 Discussion

There are various opinions and treatment options on spontaneous hemarthrosis of the knee joint in elderly patients. Causes of spontaneous hemarthrosis include subchondral bone bleeding, lateral meniscus injury, genicular artery bleeding, and the use of anticoagulants.^[2,3]^ Treatment options include conservative managements such as drug dosage adjustment and bed rest, and invasive procedures such as arthroscopic examination and cauterization.^[1]^

In 1994, Kawamura et al^[1]^ have reported a case of hemarthrosis which was caused by peripheral arterial lesions of the posterior horn of the lateral meniscus and surgically managed by arthroscopic resection of the lateral meniscal flap tear. They have also reported that most of the patients with hemarthrosis of the knee joint showed degenerative changes of the lateral compartment on plain radiographs.

Lim et al^[2]^ have also reported a case of hemarthrosis which was managed surgically by arthroscopic procedures. They have thought the cause of hemarthrosis as degenerative changes of the genicular artery and subchondral bone bleeding.

Nomura et al^[3]^ have reported that the focus of bleeding was arthroscopically revealed as the inferior lateral genicular artery in most of the elderly patients with hemarthrosis. Such lesions were managed with arthroscopic cauterization.

Conservative management should be considered prior to invasive procedures for treatment of knee joint hemarthrosis. In our case, despite the initial conservative management by discontinuing antiplatelet agents and bed resting, the degree of hemarthrosis deteriorated after the patient has begun to walk. The initial arthroscopic examination performed at a local clinic was not able to reveal any intra-articular foci of bleeding. Although arthroscopic diagnosis and treatment have been reported to be effective in a considerable amount of literature, repeating a procedure which was previously unsuccessful was demanding for the surgeons.

Many literatures have pointed out the degenerative changes of the inferior genicular artery as the cause of knee joint hemarthrosis in elderly patients.^[3]^ Likewise, since plain radiographs showed degenerative changes in the lateral compartment, we suspected that the hemarthrosis was caused by degenerative changes of genicular arteries. Angiogram, which was performed after consultation with the radiology department, showed hypervascularity of the genicular artery (Fig. 3). It was followed byone-step angiographic embolization.

Through this case, we have experienced that degenerative changes of the genicular arteries may be a cause of spontaneous hemarthrosis of the knee joint in the elderly patients, and angiography may be an effective method among the diagnostic and therapeutic options.

## Author contributions

**Conceptualization:** Won Kee Choi.

**Investigation:** Dae won Kang, Seung Bum Chae.

**Supervision:** Won kee Choi.

**Writing – original draft:** Won Kee Choi, Suk Kyoon Song.
